# Supplementary material for: An integrated resource for functional and structural connectivity of the marmoset brain
Source: Nat Commun. 2022 Dec 1;13:7416. doi: 10.1038/s41467-022-35197-2 (PMC9715556; doi:10.1038/s41467-022-35197-2)
Supplement: Supplementary file 3 — Reporting Summary [file 41467_2022_35197_MOESM3_ESM.pdf]

nature portfolio

Corresponding author(s): Cirong Liu

Last updated by author(s): Nov 8, 2022

Reporting Summary

Nature Portfolio wishes to improve the reproducibility of the work that we publish. This form provides structure for consistency and transparency in reporting. For further information on Nature Portfolio policies, see our Editorial Policies and the Editorial Policy Checklist.

Statistics

For all statistical analyses, confirm that the following items are present in the figure legend, table legend, main text, or Methods section.

n/a	Confirmed	
☐	☒	The exact sample size (*n*) for each experimental group/condition, given as a discrete number and unit of measurement
☐	☒	A statement on whether measurements were taken from distinct samples or whether the same sample was measured repeatedly
☐	☒	The statistical test(s) used AND whether they are one- or two-sided<br>*Only common tests should be described solely by name; describe more complex techniques in the Methods section.*
☐	☒	A description of all covariates tested
☐	☒	A description of any assumptions or corrections, such as tests of normality and adjustment for multiple comparisons
☐	☒	A full description of the statistical parameters including central tendency (e.g. means) or other basic estimates (e.g. regression coefficient) AND variation (e.g. standard deviation) or associated estimates of uncertainty (e.g. confidence intervals)
☐	☒	For null hypothesis testing, the test statistic (e.g. *F*, *t*, *r*) with confidence intervals, effect sizes, degrees of freedom and *P* value noted<br>*Give P values as exact values whenever suitable.*
☒	☐	For Bayesian analysis, information on the choice of priors and Markov chain Monte Carlo settings
☒	☐	For hierarchical and complex designs, identification of the appropriate level for tests and full reporting of outcomes
☒	☐	Estimates of effect sizes (e.g. Cohen's *d*, Pearson's *r*), indicating how they were calculated

Our web collection on statistics for biologists contains articles on many of the points above.

Software and code

Policy information about availability of computer code

Data collection	All MRI marmoset data were collected using Bruker's ParaVision V.6.0.1.
Data analysis	Data analysis was performed using FSL (FMRIB Software Library v5.1, Oxford University, Oxford UK), AFNI (v18.0.11, Analysis of Functional NeuroImages, National Institutes of Health, Bethesda, USA), ANTs (v2.1,Advanced Normalization Tools, University of Pennsylvania, Philadelphia, USA), TORTOISE (v3.1, National Institute of Biomedical Imaging and Bioengineering), Mrtrix3 (v3,http:// www.mrtrix.org/), Connectome Workbench for GIFTI and CIFTI data (Workbench 1.5.0)

For manuscripts utilizing custom algorithms or software that are central to the research but not yet described in published literature, software must be made available to editors and reviewers. We strongly encourage code deposition in a community repository (e.g. GitHub). See the Nature Portfolio guidelines for submitting code & software for further information.

Data

Policy information about availability of data

All manuscripts must include a data availability statement. This statement should provide the following information, where applicable:

- Accession codes, unique identifiers, or web links for publicly available datasets
- A description of any restrictions on data availability
- For clinical datasets or third party data, please ensure that the statement adheres to our policy

All of our datasets, including raw and preprocessed NIH and ION resting-state fMRI, diffusion MRI, and neuronal tracing datasets, are available on our specific webpage of the Marmoset Brain Mapping Project (www.marmosetbrainmapping.org/data.html). The volume data are in NIFTI format, and the surface data are in CIFTI format. The raw MRI data without processing is provided in the standard BIDS format for cross-platform sharing. The MBMv4 parcellations are also provided in the same webpage (www.marmosetbrainmapping.org/data.html), and the MBMv1 and Paxinos parcellations on our MRI template space are a part of the MBMv3

resource (marmosetbrainmapping.org/v3.html). The high-resolution ex-vivo diffusion MRI data are a part of the MBMv2 resource (marmosetbrainmapping.org/atlas.html#v2). The raw neuronal tracing data are from Marmoset Brain Connectivity project (https://www.marmosetbrain.org/reference). Note that the MBMv4 datasets are only available for scientific purposes and are licensed under Creative Commons Attribution-NonCommercial-ShareAlike (CC BY-NC-SA 4.0).

Field-specific reporting

Please select the one below that is the best fit for your research. If you are not sure, read the appropriate sections before making your selection.

☒ Life sciences ☐ Behavioural & social sciences ☐ Ecological, evolutionary & environmental sciences

For a reference copy of the document with all sections, see nature.com/documents/nr-reporting-summary-flat.pdf

Life sciences study design

All studies must disclose on these points even when the disclosure is negative.

Sample size	The study aimed to provide a comprehensive MRI data resource for marmoset brain mapping and thus the sample size should be as large as possible. Generally, the minimum sample for population-level analysis should be no less than 10. In the study, we recruited a total of 39 marmosets from two institutes which constitutes the largest awake resting-state fMRI database for marmosets. The sample size is proved to be sufficient, because the population-level brain parcellations are converged to produce similar parcellation results for two institutes.
Data exclusions	For each fMRI run, any time points and the previous time points were censored if the detection motion was > 0.2 mm. The motion censor is commonly used in fMRI preprocess to reduce the influence of head motions.
Replication	We performed replication of brain parcellation at the population-level (across-sites) and individual level. At the population level, the replication reached a high dice index of 0.7, indicating a common brain parcellation pattern across animals. At the individual level, the replications had indices less than 0.4, demonstrating the large individual variability in resting-state functional connectivity.
Randomization	All animals were tested one-by-one against the population results and thus no randomization were needed.
Blinding	All animals were normal subjects and belonged to one health group. Blinding were not applied to this study.

Reporting for specific materials, systems and methods

We require information from authors about some types of materials, experimental systems and methods used in many studies. Here, indicate whether each material, system or method listed is relevant to your study. If you are not sure if a list item applies to your research, read the appropriate section before selecting a response.

Materials & experimental systems

n/a	Involved in the study	
☒	☐	Antibodies
☒	☐	Eukaryotic cell lines
☒	☐	Palaeontology and archaeology
☐	☒	Animals and other organisms
☒	☐	Human research participants
☒	☐	Clinical data
☒	☐	Dual use research of concern

Methods

n/a	Involved in the study	
☒	☐	ChIP-seq
☒	☐	Flow cytometry
☐	☒	MRI-based neuroimaging

Animals and other organisms

Policy information about studies involving animals; ARRIVE guidelines recommended for reporting animal research

Laboratory animals	We recruited 39 common marmosets (Callithrix jacchus, in age ranges of 2 to 9 year old). Thirteen marmosets (12 males and 1 female) were recruited from the ION cohort and Twenty-six marmosets (19 males and 7 females) were recruited from the NIH cohort.
Wild animals	this study did not involve wild animals.
Field-collected samples	The study did not involve samples collected from the field.
Ethics oversight	The experimental procedures were approved by the Laboratory Animal Care and Use Committees from the Institute of Neuroscience (ION) at the Chinese Academy of Sciences and National Institute of Neurological Disorders and Stroke at the National Institutes of Health (NIH).

Note that full information on the approval of the study protocol must also be provided in the manuscript.

Magnetic resonance imaging

Experimental design

Design type

Resting-state fMRI and diffusion MRI

Design specifications

Each resting-state fMRI run had 17 mins, each session included 4 - 8 runs, and each marmoset were scanned 1 - 10 sessions. One diffusion MRI session were scanned for 25 marmosets.

Behavioral performance measures

n/a (resting-state fMRI and diffusion MRI didn't involve behavioral performance measures)

Acquisition

Imaging type(s)

functional MRI and diffusion MRI

Field strength

7T and 9.4T

Sequence & imaging parameters

The ION marmosets were scanned in a 9.4T/30cm horizontal MRI scanner (Bruker, Billerica, USA) equipped with a 20 cm gradient set capable of 300 mT/m gradient strength. The scanner was fitted with a 154 mm ID quadrature RF coil used for signal excitation and an 8-channel phased-array RF coil custom-built for marmosets (Fine Instrument Technology, Brazil). Multiple runs of rs-fMRI data were collected in ParaVision 6.0.1 software using a 2D gradient-echo (GE) EPI sequence with the following parameters: TR=2 s, TE=18 ms, flip angle=70.4°, FOV=28 ×36mm, matrix size=56×72, 38 axial slices, slice thickness=0.5 mm, 512 volumes (17min) per run. The GE-EPI fMRI data were collected using two opposite phase-encoding directions (LR and RL) to compensate for EPI distortions and signal dropouts. Two sets of spin-echo EPI with opposite phase-encoding directions (LR and RL) were also collected for EPI-distortion correction (TR= 3000ms, TE=37.69 ms, flip angle=90°, FOV=28 ×36 mm, matrix size=56×72, 38 axial slices, slice thickness=0.5 mm, 8 volumes for each set). After each rs-fMRI session, a T2-weighted structural image (TR=8000 ms, TE=10 ms, flip angle= 90°, FOV= 28 ×36mm, matrix size=112 ×144, 38 axial slices, slice thickness=0.5mm) was scanned for co-registration purposes.

The NIH marmosets were scanned in a 7T/30cm horizontal MRI (Bruker, Billerica, USA) equipped with a 15 cm customized gradient set capable of 450 mT/m gradient strength (Resonance Research Inc., Billerica, USA). The scanner was fitted with a 110 mm ID linear RF coil used for signal excitation and an 8-channel phased-array RF coil custom-built for marmosets. During each scanning session, multiple runs of rs-fMRI data were collected in ParaVision 6.0.1. software using a 2D gradient-echo (GE) EPI sequence with the following parameters: TR=2s, TE=22.2ms, flip angle=70.4°, FOV= 28×36mm, matrix size=56×72, 38 axial slices, slice thickness=0.5mm, 512 volumes (17min) per run. The GE-EPI fMRI data were collected using two opposite phase-encoding directions (LR and RL) to compensate for EPI distortions and signal dropouts. Two sets of spin-echo EPI with opposite phase-encoding directions (LR and RL) were also collected for EPI-distortion correction (TR=3000ms, TE=36ms, flip angle = 90°, FOV=28 × 36mm, matrix size = 56 × 72, 38 axial slices, slice thickness=0.5mm, 8 volumes for each set). After each rs-fMRI session, a T2-weighted structural image (TR= 6000ms, TE=9ms, flip angle=90°, FOV=28×36mm, matrix size = 112×144, 38 axis slices, slice thickness=0.5mm) was scanned for co-registration purposes.

Area of acquisition

whole brain

Diffusion MRI ☒ Used ☐ Not used

Parameters

Multishell diffusion MRI (DTI) datasets were collected using a 2D diffusion-weighted spin-echo EPI sequence with the following parameters: TR = 5.1 s, TE = 38 ms, number of segments = 88, FOV = 36 × 28 mm, matrix size = 72 × 56, slice thickness = 0.5 mm, a total of 400 DWI images for two-phase encodings (blip-up and blip-down).and each has 3 b values (8 b = 0, 64 b =2400, and 128 b = 4800), and the scanning duration was about 34 min. The multishell gradient sampling scheme was generated using the Q-shell sampling method.

Preprocessing

Preprocessing software

Preprocessing involves the following software:
FSL (FMRIB Software Library v5.1, Oxford University, Oxford UK),
AFNI (v18.0.11, Analysis of Functional NeuroImages, National Institutes of Health, Bethesda, USA),
ANTs (v2.1,Advanced Normalization Tools, University of Pennsylvania, Philadelphia, USA), TORTOISE (v3.1, National Institute of Biomedical Imaging and Bioengineering).

Normalization

The preprocessed data were spatially normalized to the template space of our Marmoset Brain Atlas Version-3 (MBMv3) by the "antsRegistration" routine of ANTs. The spatial normalization concatenated multiple transformations, including 1) rigid-body transformation of each fMRI run to the T2-weighted image acquired at the end of each session, 2) rigid-body transformation of T2-weighted images from each session to a cross-session averaged T2-weighted image from each animal, 3) affine and nonlinear transformation of the averaged T2-weighted image from each animal to the T2w template of our MBMv3 space.

Normalization template

We normalized the fMRI data to the Marmoset Brain Mapping Version 3 (MBMv3) template space.

Noise and artifact removal

The rs-fMRI datasets were further preprocessed by regressing linear and quadratic trends, demeaning, and censoring for motion using derivatives of motion parameters and motion-sensor regressors (any TRs and the previous TRs were censored if the detection motion was > 0.2 mm). White matter and cerebrospinal fluid signal were removed, and the rs-fMRI datasets were band-pass filtered (0.01–0.1 Hz). The above nuisance signal regression and band-passing filtering were carried out by the "3dDeconvolve" and "3dTproject" commands in AFNI.

Volume censoring

For each fMRI run, any time points and the previous time points were censored if the detection motion was > 0.2 mm. The motion censor is performed as an reg

Statistical modeling & inference

Model type and settings

Multivariate and univariate

Effect(s) tested

We used multi-comparison one-way ANOVA test for the difference functional parcellations and mapping results; We used Wilcoxon paired signed-rank test for the task-fMRI activation consistency. We used Wilcoxon rank sum test for the MRI data signal-of-noise ratio from different scanners

Specify type of analysis: ☐ Whole brain ☐ ROI-based ☒ Both

Anatomical location(s)

The anatomical locations were defined based our MBMv4 atlas developed in this study and previously published atlases in MBMv3 template space, including MBMv1 parcellations, the RIKEN atlas and the Paxinos atlas.

Statistic type for inference
(See Eklund et al. 2016)

For the task activation map in Figure 6, A mixed-effects analysis was then applied to all statistical maps across sessions by the 3dMEMA command of AFNI to obtain a final statistical map. The map was thresholded at a voxel-wise threshold of p< 0.05 and a cluster-wise threshold of p<0.05 for multiple comparison corrections.

Correction

The map was thresholded at a voxel-wise threshold of p<0.05 and a cluster-wise threshold of p<0.05 for multiple comparison corrections.

Models & analysis

n/a	Involved in the study
☐	☒ Functional and/or effective connectivity
☒	☐ Graph analysis
☐	☒ Multivariate modeling or predictive analysis

Functional and/or effective connectivity

We used the Pearson's correlation to calculate the functional connectivity and structural connectivity, as well as the similarity metric between different functional connectivity

Multivariate modeling and predictive analysis

1) We used the evaluation metric of distance-controlled boundary coefficient (DCBC) to evaluate functional boundaries from fMRI data. The rationale of the method is that if a boundary is dividing two functional heterogeneous regions, then the two voxels within the same region should have more similar functional profiles than two voxels in the different areas. Therefore, the correlation between two voxels will be higher for the two adjacent voxels and fall off as the spatial distance increases. To control for distance, we calculated the activation pattern correlations for all pairs of voxels separated by a fixed Euclidean distance, using spatial bins ranging from 0mm to 4mm. Since that, this allowed us to test for significant differences between different parcellations (such as RIEKN and Paxions atlas) using multiple comparsions for One-Way ANOVA.
2) We used a deep-learning network to map individual data from the population-based data. The network was designed as a multi-layer deep neural network, comprising three layers (one input, five hidden, one output) and 384 hidden neurons (a reasonable compromise between accuracy and training speed for classification). The whole-brain fingerprint of the candidate parcel from the proposed MBMv4 worked as training set for the network to classify whether or not each vertex in an individual ROI containing the parcel plus all of its neighbor parcels.
3) We used the Hopf modeling to link structural and functional connectivity. First, the input structural data representation is N (regions) x P (regions) data matrix according to the different individuals. Then we added every connection hub with a Hopf function. It allows us to reversely simulated the fMRI timecourses based on this structural connectivity. When we got the fMRI time courses, we could calculate the simulated functional connectivity N' (regions) x P' (regions) data matrix and its comparison with the experienced functional connectivity N'' (reigons) x P'' (regions) from actual data. The metric for the comparison in the Pearson correlation.
